# Aging Impairs Recipient T Cell Intrinsic and Extrinsic Factors in Response to Transplantation

**DOI:** 10.1371/journal.pone.0004097

**Published:** 2009-01-01

**Authors:** Hua Shen, Bethany M. Tesar, Wei Du, Daniel R. Goldstein

**Affiliations:** Department of Internal Medicine, Yale University School of Medicine, New Haven, Connecticut, United States of America; New York University School of Medicine, United States of America

## Abstract

**Background:**

As increasing numbers of older people are listed for solid organ transplantation, there is an urgent need to better understand how aging modifies alloimmune responses. Here, we investigated whether aging impairs the ability of donor dendritic cells or recipient immunity to prime alloimmune responses to organ transplantation.

**Principal Findings:**

Using murine experimental models, we found that aging impaired the host environment to expand and activate antigen specific CD8^+^ T cells. Additionally, aging impaired the ability of polyclonal T cells to induce acute allograft rejection. However, the alloimmune priming capability of donor dendritic cells was preserved with aging.

**Conclusion:**

Aging impairs recipient responses, both T cell intrinsic and extrinsic, in response to organ transplantation.

## Introduction

During the last decade, increasing numbers of people over 65 years of age are currently waiting for kidney transplants [1], [2]. Since the number of older people in our society will continue to grow and as many end stage organ disorders are associated with increased age, it is likely that an even larger number of older patients will receive solid and cellular transplants in the coming years [2], [3].

Clinical studies have indicated that older people have a reduced immune response to infections as well as an impaired ability to reject organ allografts [4]–[11]. Overall, it is not clear how aging alters the response to organ transplantation. Some experimental reports have indicated that reduced alloimmune responses may be due to declining cellular immunity [12]–[14], while other reports have shown that increasing donor age can negatively impact allograft survival [15]. In contrast to these reports, there is evidence that aging is associated with increased chronic rejection [16]. Hence, aging may impact alloimunity via multiple effects, although it is unclear whether altered cellular alloimmunity with aging is due to impaired priming by dendritic cells (DCs) or due to defective intrinsic T cell function.

Donor DCs, recipient DCs and T cells are some of the critical cellular mediators of acute allograft rejection as defects in these cells can significantly impair the ability of recipients to reject organ transplants [17]–[19]. However, the impact of aging on the effect on each of these cells in the setting of allotransplantation is not fully elucidated and it is not clear if declining cellular alloimmunity with aging is due to impaired priming by DCs or defective recipient factors intrinsic or extrinsic to the T cell. Hence, the goal of the present study was to determine whether aging impairs alloimmune priming function of donor DCs, recipient DCs or T cell intrinsic alloimmune responses. We provide evidence that factors both intrinsic and extrinsic to recipient T cells are defective with aging in response to skin transplantation, however aging does not impair the ability of donor DCs to prime alloimmune responses in our experimental systems.

## Results

### Aged donor bone marrow derived DCs (BMDCs) exhibit similar alloimmune priming capabilities as compared to young counterparts

**Figure 1:**
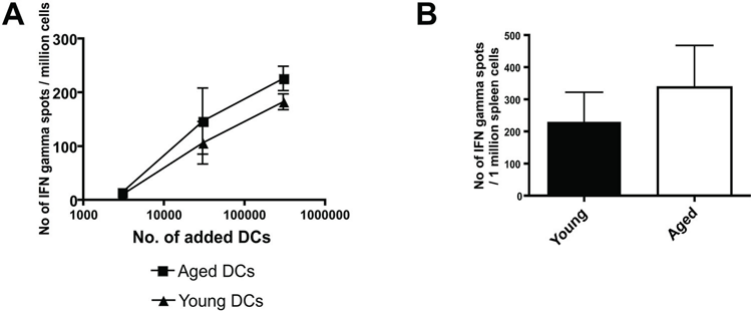
Aged BMDCs exhibit a similar alloimmune priming ability as compared to young BMDCs. A: Young C57BL/6 mice were immunized (via i.p. injection) with 1×10^7^ young CBA spleen cells. After two weeks, recipient spleen cells were harvested and cultured *ex vivo* overnight with increasing numbers of either aged or young irradiated CBA BMDCs and IFN-γ responses measured (ELISPOT). B: Aged or young CBA BMDCs were i.p. injected into young C57BL/6 mice. Two weeks later, recipient spleen cells were harvested and cultured *ex vivo* overnight with young irradiated CBA spleen cells. IFN-γ responses measured via ELISPOT. Similar results were obtained when the re-stimulating irradiated donor spleen cells were aged (data not shown).

Prior work has indicated that donor DCs are one of the main cellular activators of acute allograft rejection [20]. Hence, we examined the effect of age on the ability of BMDCs to stimulate allo-reactive splenocytes. Young C57BL/6 mice were immunized with young CBA spleen cells. Two weeks later, recipient spleen cells were harvested and stimulated *ex vivo* with irradiated aged (20–22 months of age) or young (2–4 months of age) CBA BMDCs. The results show that aged BMDCs induced similar production of IFN-γ in allo-reactive spleen cells as compared to young BMDCs (Figure 1A). These results are consistent with prior work that demonstrated that aged and young allogeneic BMDCs were equally able to activate naïve T cells in vitro [21].

To examine the role of aging on the *in vivo* priming capabilities of allogeneic BMDCs, aged or young CBA BMDCs were injected into young C57BL/6 mice. Two weeks later the recipient spleens were harvested and stimulated *ex vivo* with young, irradiated CBA spleen cells. The results demonstrate that aged donor BMDCs exhibit a similar *in vivo* priming ability, as measured by IFN-γ produced by recipient spleen cells, compared to young counterparts (Figure 1B).

### Splenic APCs and DCs exhibit a preserved ability to prime allogeneic T cells with aging

**Figure 2:**
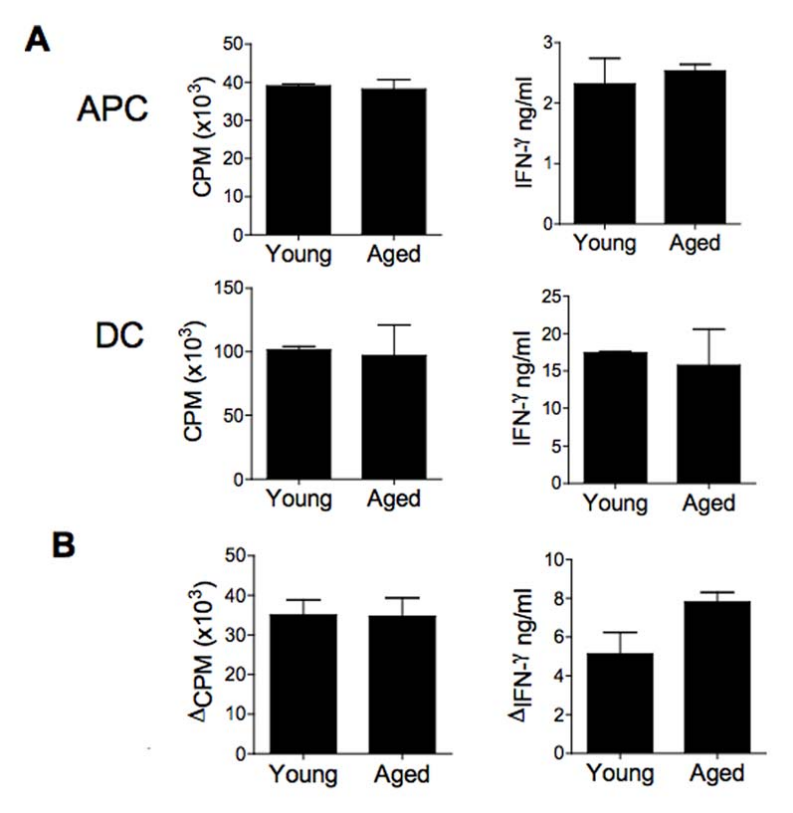
Aged splenic DCs and APCs exhibit a similar ability to prime allogeneic T cell responses both in vitro and in vivo. A: Splenic APCs and CD11c^+^ DCs, purified from aged or young CBA mice, were cultured with C57BL/6 T cells and cellular proliferation and IFN-γ measured. Similar results were noted in two independent experiments and with an alternate donor (C57BL/6) and recipient T cell (BALB/C) combination. B: Aged or young splenic CBA DCs were injected into C57BL/6 mice. 10 days later spleens were harvested from the groups and cultured with irradiated CBA spleen cells, and cellular proliferation and IFN-γ measured. The change from T cells from mice that were not immunized is shown. There were no significant differences noted between mice injected with either young or aged allogeneic DCs. Representative data from one experiment, which was repeated with consistent results.

In the above experiments, we employed BMDCs, which were expanded *ex vivo* with GM-CSF. As this may have masked a defect with aging, we next performed experiments with antigen presenting cells (APCs) and DCs that were purified directly from mice, without any further *ex vivo* expansion. Splenic APCs and DCs purified from aged (18–20 months of age) mice exhibited a similar ability to induce allogeneic T cell proliferation and IFN-γ production *in vitro* as compared to young cells (Figure 2A). Furthermore, DCs purified from aged mice exhibited a similar alloimmune priming response *in vivo* (Figure 2B) as compared to young cells. Additionally, aged DCs manifested similar surface expression of MHC class II, CD40, CD54 and CD86 at rest and after culture with allogeneic T cells as compared to young DCs (data not shown).

### Graft originating APCs exhibit a similar ability to prime allogeneic T cell responses and induce a similar tempo of allograft rejection with aging

**Figure 3:**
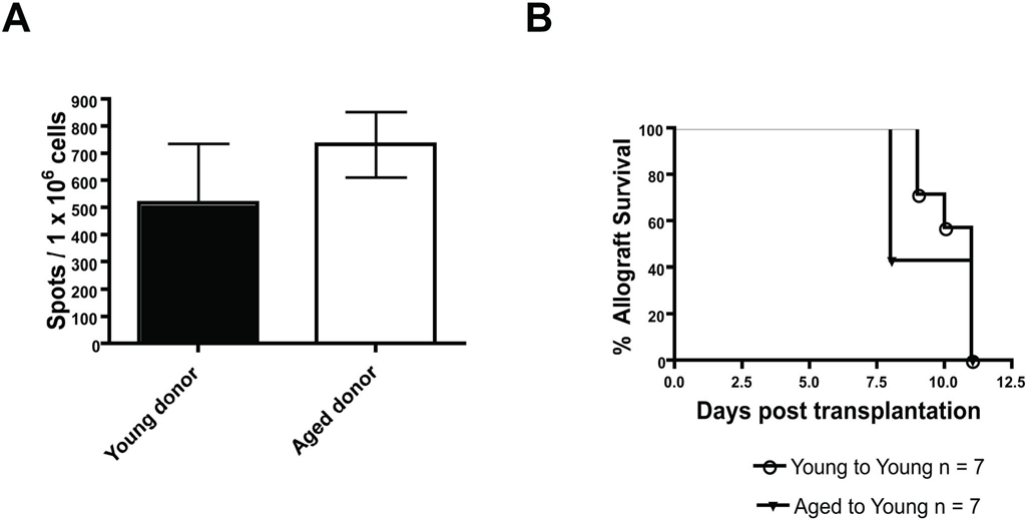
Aging does not impair the ability of graft originating APCs to prime alloimmunity. C: Young CBA recipients received either aged or young C57BL/6 skin allografts. Two weeks after transplantation, recipient spleens were removed and then restimulated overnight in the presence of irradiated donor spleen cells. (No differences were noted if aged or young donor irradiated stimulators were used, data not shown). D: Young CBA recipients were transplanted with either an aged or young C57BL/6 skin allograft and the time to rejection was monitored. Young recipients of aged allografts manifest similar rejection kinetics compared to young counterparts that received young allografts (p = 0.5; Log rank).

To investigate whether aging influenced the ability of graft originating, donor APCs to prime alloimmune responses, aged or young C57BL/6 allografts were transplanted onto *young* CBA recipients. In this experimental design, all the recipient DCs and T cells are young and the experimental variable is the age of the donor APC. Two weeks later, recipient spleen cells were harvested and stimulated *ex vivo* with irradiated donor (i.e., C57BL/6) spleen cells. The results show that aged allografts induced similar IFN-γ responses in young recipients as compared to young recipients transplanted with a young allograft (Figure 3A). Finally, aged C57BL/6 allografts underwent a similar tempo of allograft rejection when transplanted onto young CBA recipients as compared to young allografts transplanted onto young recipients (Figure 3B). In sum, these data indicate that aging does not impair the ability of donor, graft originating APCs to prime T cell responses in our experimental systems.

### The aged host environment impairs the activation and expansion of transplant specific CD8^+^ T cells

We next examined the impact of aging on recipient APCs to prime and expand alloreactive T cells. Hence, aged or young CD45.2^+^, Thy1.2^+^ C57BL/6 mice were adoptively transferred with *young* naïve (CD44^lo^, CD62L^hi^) CD45.1^+^ TCR transgenic T cells that are reactive to the SINFEKL OVA_257–264_ peptide presented by MHC class I (cells denoted as OTI) and *young* naïve Thy1.1^+^ transgenic T cells that are reactive to OVA_323–339_ peptide presented by MHC class II (cells denoted as OTII). Two days later, aged or young adoptively transferred mice received a young B6 OVA-expressing skin transplant (Act-mOVA). These mice express a membrane-bound form of OVA that is expressed under the control of β-actin promoter. This is an established minor-mismatched transplant model, which allows one to track the fate of graft-specific T cells in vivo [22]. Thus, in this experimental design the variable is the recipient age, which contains recipient APCs.

**Figure 4:**
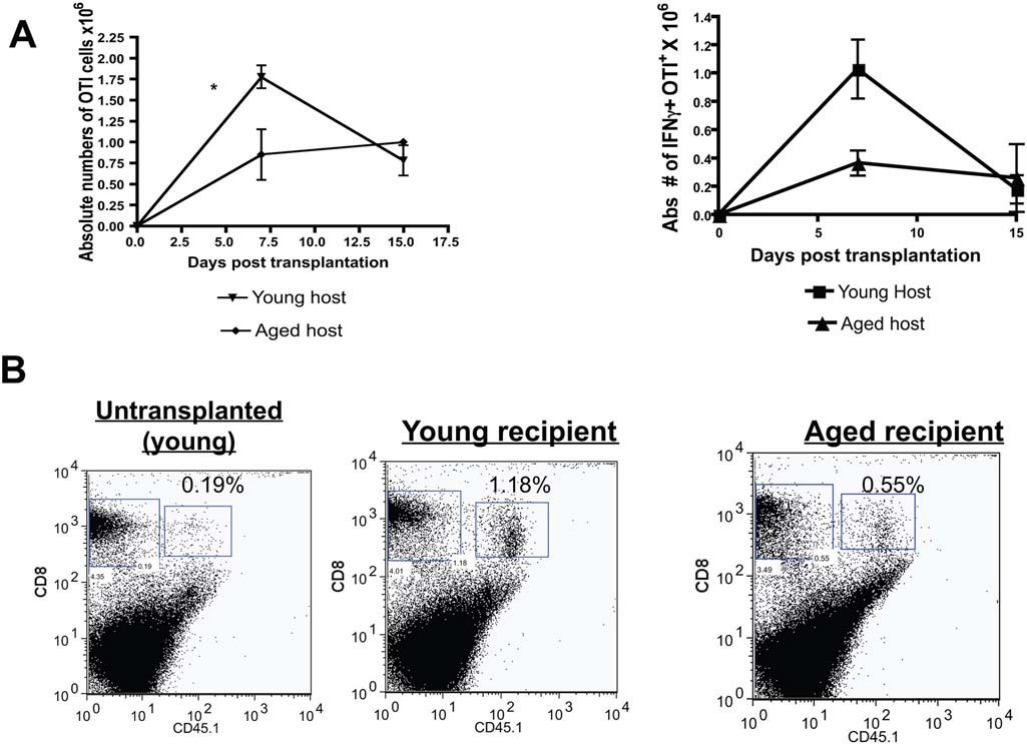
Recipient age impairs the expansion of transplant reactive CD8^+^ T cells. C57BL/6 aged and young CD45.2^+^, Thy1.2^+^ mice were adoptively transferred with 5×10^5^ CD45.1^+^, OTI T cells 2 days prior to receiving a B6.Act-mOVA skin transplant. A: At various points after transplantation, recipient spleen cells were harvested and the number of OTI T cells or OTI T cells that produced IFN-γ calculated. Expansion of transplant/antigen specific T cells was reduced in an aged environment as compared to a young environment. (*P<0.02; T test at day +7 post transplantation). Representative data of three independent experiments with N = 2–3/experiment. B: Representative flow cytometric dot plots are shown at day +7 post transplantation in young and aged mice. A representative, non-transplanted recipient that received OTI T cells is shown. The transferred cells (CD45.1^+^, CD8^+^) are shown in the rectangle. Representative data of three independent experiments with N = 2–3/experiment.

**Figure 5:**
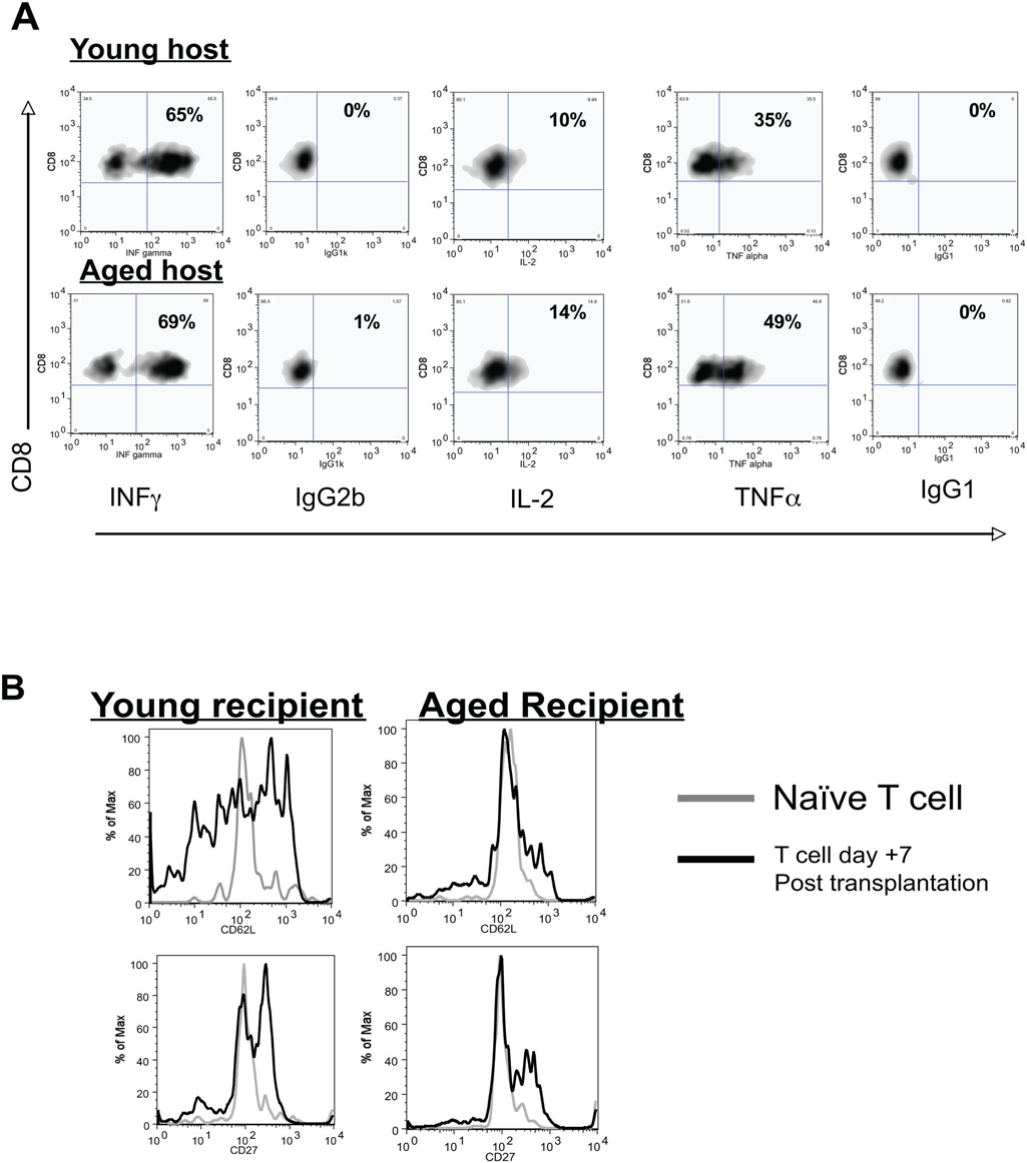
Transplant specific CD8^+^ T cells produced similar cytokine profiles but impaired activation in aged transplant recipients as compared to young recipients. A: Representative flow cytometric plots on day +7 post transplantation are shown along with isotype controls (IgG2b for IFNγ, IgG1 for IL-2 and TNFα). Young antigen specific CD8^+^ T cells produced similar amounts of TNFα, IL-2 and IFN-γ during *ex vivo* culture with OVA peptide regardless of the age of the recipient environment (cytokines were not produced if peptide was not added, data not shown). Proportions are shown in right upper quadrant. Note, that cytokines were not detected in either aged or young mice that were adoptively transferred but did not receive an OVA expressing skin transplant (data not shown). Similar results were noted at day +14 post transplantation (data not shown). Flow cytometric plots are gated on CD45.1^+^ cells. N = 3/group, representative data from three independent experiments with consistent results. B: Representative flow cytometric plots of adoptively transferred CD45.1^+^, OTI young T cells on day +7 post transplantation. Young antigen specific CD8^+^ T cells upregulated CD27 and downregulated CD62L to a greater degree in young transplant recipients compared to antigen specific CD8^+^ T cells in aged transplant recipients.

At various time points post transplantation, spleen cells were harvested and the cytokine function and expansion of young OTI (CD8^+^) and OTII (CD4^+^) T cells in either aged or young recipients were measured. We found that the peak expansion of young antigen specific CD8^+^T cells was reduced in an aged environment as compared to a young environment (Figure 4). However, young antigen specific CD8^+^T cells produced similar cytokine responses for IFN-γ, IL-2 and TNFα regardless of the age of the host environment (Figure 5A). Nevertheless, the activation status (measured by the up-regulation of CD27 and down-regulation of CD62L) of antigen specific CD8^+^ T cells was higher in young hosts than in aged hosts (Figure 5B). We noted that CD4^+^ T cell responses were generally a log-fold lower than CD8^+^ T cell responses in this experimental model and with similar responses in either an aged or young environment (data not shown). These data indicate that aging impairs the recipient host environment (containing APCs) to activate and expand antigen specific CD8^+^ T cell responses in vivo, although the aged host environment allows effective cytokine responses in these T cells.

**Figure 6:**
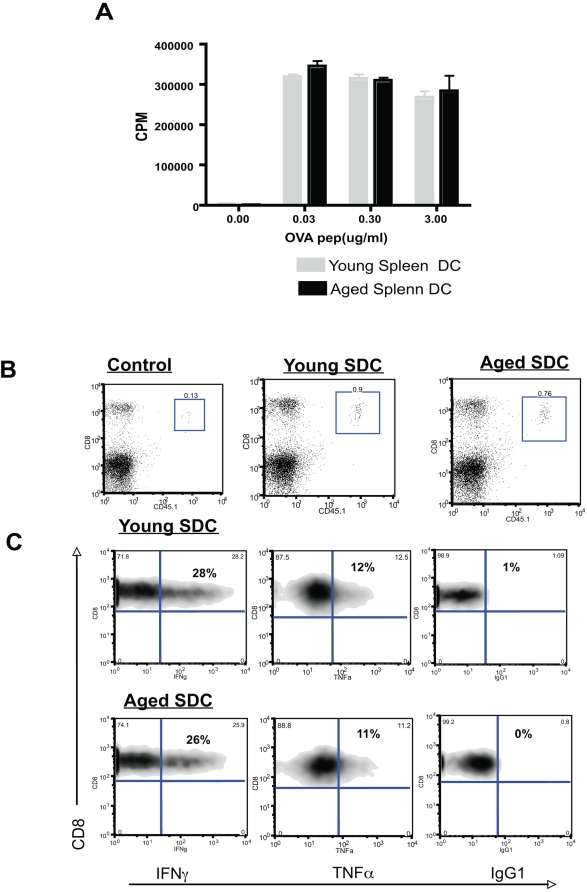
Aged splenic DCs loaded with cognate peptide exhibit a similar ability to prime antigen specific CD8^+^ T cells as compared to young splenic DCs. A: Aged and young splenic DCs were cultured with OTI T cells in the presence of OVA peptide and proliferation measured. Representative from one experiment, which was repeated with similar results. B: Young hosts (N = 3/group) were adoptively transferred with young CD45.1^+^ OTI T cells and either young or aged splenic DCs that had been pulsed with OVA peptide (or irrelevant peptide, GP33, as a control). After 5 days, expansion of transferred young OTI T cells was assessed. Proportion of transferred T cells is shown in the box in each flow plot. Representative data from one experiment, which was repeated with similar results. C: Same experiment as B except that cytokine production by the transferred OTI T cells (gated on CD45.1^+^ population) is measured by intracellular cytokine staining. Proportion % is shown in each flow plot.

The above results indicate that factors within the aged host extrinsic to the T cell impair the expansion of antigen specific CD8^+^ T cells. To examine if aging impaired the ability of DCs to process and present cognate peptides to antigen specific CD8^+^ T cells, we measured the ability of young or aged splenic DCs to activate OTI CD8^+^ T cells *in vitro*. Our results demonstrate that aged splenic DCs, cultured with OVA peptides, were able to induce a similar degree of proliferation in OTI CD8^+^ T cells in comparison to young splenic DCs (Figure 6A). We next examined the ability of aged and young splenic DCs, which were pulsed with OVA peptides and then adoptively transferred into young syngeneic recipients, to expand and activate OTI CD8^+^ T cells *in vivo*. The results demonstrate that OTI T CD8^+^ T cells transferred with aged splenic DCs into young recipients exhibited a similar expansion and inflammatory cytokine response as compared to young OTI CD8^+^ T cells transferred with young splenic DCs into young recipients (Figure 6B–C).

### Aging impairs T cell responses to allotransplantation

**Figure 7:**
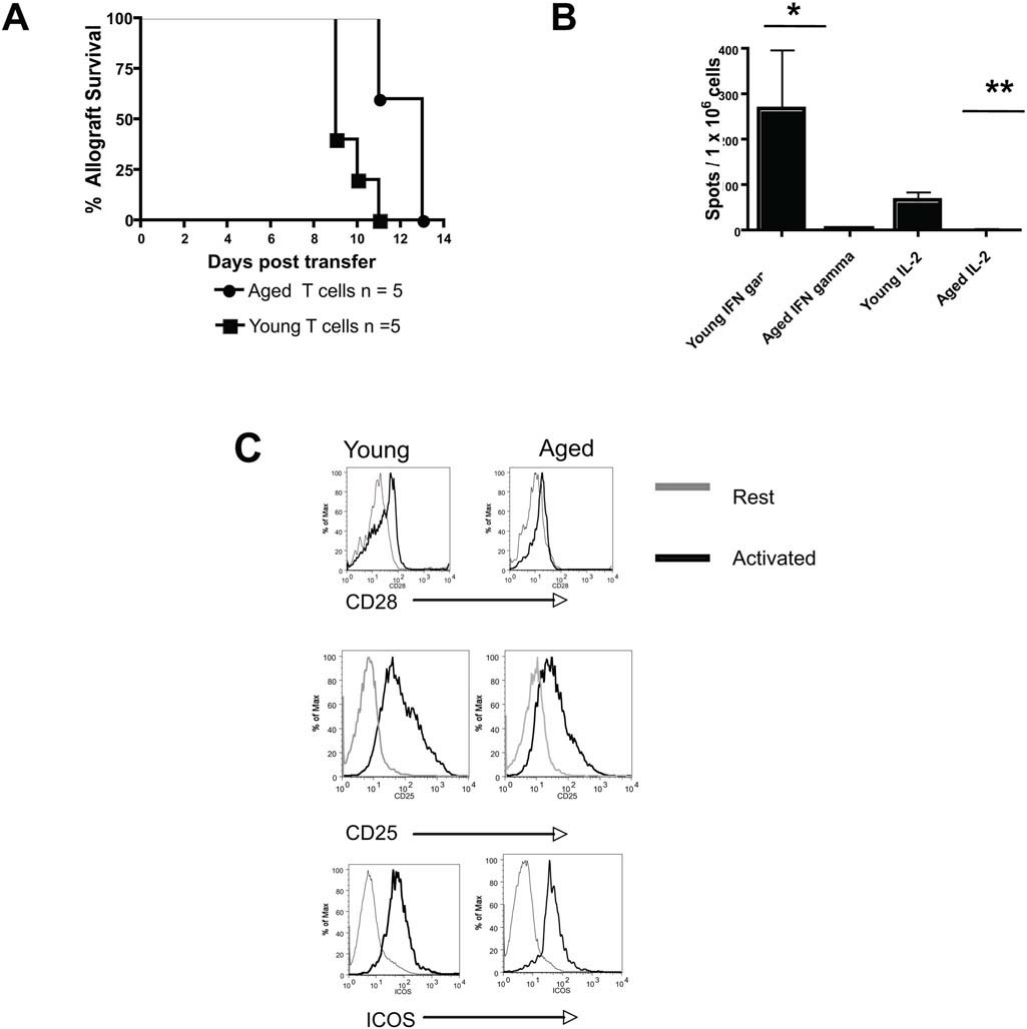
Aging impairs T cell alloimmune responses. A: Young C57BL/6 T cell deficient mice reconstituted with 5×10^6^ aged (20 mths) syngeneic, polyclonal T cells rejected BALB/c skin allografts at a slower tempo than counterparts reconstituted with young (2–4 mths) T cells. (*p = 0.007; Log rank). B: Aged or young CBA recipients were transplanted with young C57BL/6 skin transplants. At two weeks post transplantation, recipient spleen cells were harvested and CD4^+^ T cells purified. The results demonstrate that aged CD4^+^ T cells that were stimulated ex vivo with irradiated donor spleen cells manifest impaired IFNγ (*p<0.0001; T test) and IL-2 (**p<0.0001; T test) responses (ELISPOT) compared to young CD4^+^ T cells. N = 3/group, representative data from two independent experiments with consistent results. C: Aged and young purified, polyclonal T cells were stimulated with C57BL/6 BMDCs and the upregulation of indicated activation markers shown after 96 h of culture. N = 3/group, representative data three independent experiments with consistent results.

Our results above indicate that aging does not impair the ability of either donor or recipient DCs to prime transplant specific T cell responses. We next examined if aging impairs T cell alloimmune responses, by adoptively transferred magnetically purified aged or young T cells into young T cell deficient mice, which received a skin allograft two days earlier. We found that young recipients that received aged T cells manifested a slightly delayed time to allograft rejection as compared to young recipients that received young T cells (Figure 7A).

To examine whether aging affects recipient T cell responses primed by young, donor APCs *in vivo*, young or aged CBA recipients were transplanted with a young C57BL/6 allograft. Two weeks post transplantation, recipient T cells were enriched and CD4^+^ T cells purified via magnetic sorting. The purified CD4^+^ T cells, which we confirmed by flow cytometry were devoid of recipient APCs (data not shown), were subsequently stimulated ex vivo with irradiated donor spleen cells. The results show that aging significantly impaired the ability of CD4^+^ T cells to respond to young donor APCs as compared to young CD4^+^ T cells (Figure 7B).

Finally, in vitro stimulation with allogeneic BMDCs, revealed that aged polyclonal T cells did not upregulate certain T cell activation markers, CD25 and CD28, to the same degree as young polyclonal T cells, although the upregulation of other activation markers, e.g., ICOS, was preserved with aging (Figure 7C). Prior work has shown that aged T cells manifest impaired IL-2 and IFN-γ responses in this assay [21]. In summary, the above data indicate that aging impairs T cells to respond to allostimulation.

## Discussion

Aging has been associated with lower frequencies of acute allograft rejection, yet it has not been clear if such a phenotype is due to impaired donor or recipient APC priming of alloreactive T cells. Our study has provided evidence that aging impairs the ability of the recipient host environment, both intrinsic and extrinsic to the T cell, to respond to skin transplants. However, the alloimmune priming capabilities of donor APCs are preserved with aging in our experimental systems. Other work has indicated that aging augments immune priming by donor DCs [23] and our study does not exclude that aging may play important roles of the function of donor DCs, especially when DCs originate from vascularized allografts. Overall, our study indicates that aging impairs recipient immune responses to allostransplantation, whereas donor APC and DC responses remain preserved in our experimental systems.

We determined that aging also impaired the ability of the recipient host environment, extrinsic to the T cell, to expand and activate antigen specific naïve CD8^+^ T cells in response to skin transplantation (Figure 2 and 3B). We directly examined whether aging led to a defective ability of recipient splenic DCs to prime and activate antigen specific T cells. In our experimental system, the antigen in this case, an OVA peptide, was also a transplant antigen. However, we did not discern that aged splenic DCs exhibited a defective ability to expand and activate antigen specific CD8^+^ T cells as compared to young DCs. It is not clear what factors within the aged host impair the ability of antigen specific CD8^+^ T cells to expand, although possibilities include heightened BCL-2 and type I interferon levels. This is because a prior study, which employed non-transplant experimental models, found that the aged host environment impaired the turnover of memory CD44^hi^ CD8^+^ T cells via heightened BCL-2 expression and higher type I interferon levels [24]. Future studies are warranted to determine the factors within the aged host environment that impair the expansion of antigen-specific, naïve CD8^+^ T cells.

In conclusion, our study provides evidence that aging impairs recipient factors, both intrinsic and extrinsic to the T cell during transplantation. This information may provide a foundation for further mechanistic studies investigating how aging modifies alloimmunity, as well as translational studies that investigate how older people respond differently to solid organ allografts.

## Materials and Methods

### Mice

Aged (18–22 months) and young (2–4 months) CBA (H2^k^) and C57BL/6 (H2^b^) mice were purchased from the NIA rodent facility. B6.129P2-Tcrb^tm1Mom^Tcrd^tm1Mom^ (designated as T cell deficient mice), BALB/c and B6 Act-mOVA mice were purchased from the Jackson Laboratories, Bar Harbor, ME. B6.CD45.1^+^ OT1 and B6.Thy1.1^+^ OTII mice were generously provided by Dr. Richard Flavell and Dr. Lauren Cohn, respectively (Yale University). Yale University IACUC approved the use of animals in this study. All mice were kept in pathogen free conditions. No animals were used in the study if they had evidence of skin lesions, weight loss or lymphadenopathy. Additionally, sentinel mice were regularly tested by Yale Rodent Services and found to be negative for common murine pathogens in the serum, nasopharynx and the gastrointestinal tract (pathogens tested included mouse hepatitis virus, parvovirus, mycoplasma pneumonia, sendai virus, lymphocytic choriomengitis virus, ectromelia virus, pneumonia virus and epizootic diarrhea of infant mice).

### Skin transplantation

Full-thickness trunk skin was transplanted from donor mice and stapled on recipients as previously described [25]. Rejection was defined as graft necrosis >90% of the graft area.

### Cell purification, and *in vitro* T cell cultures

The generation of BMDCs has been described in our previous work [26]. Splenic DCs were purified by positive selection (anti-phycoerythrin-CD11c monoclonal antibody) using EasySep (StemCell Technologies, Vancouver, BC, Canada). Splenic APCs were purified by depleting spleen cells of T cells, using reagents from EasySep. T cells were purified via negative magnetic selection using EasySep reagents. To perform the primary mixed lymphocyte reaction (MLR), 1×10^5^ purified CBA T cells/well were cultured with irradiated (28Gy) allogeneic C57BL/6 BMDCs (1×10^5^/well), splenic DCs (1×10^4^/well), splenic APCs or whole splenic cells (1×10^5^/well) and incubated in 96 well plates at 37°C in a 5% CO_2_ incubator for 4 days. To perform *ex vivo* analysis from immunized mice, T cells were cultured with the indicated irradiated donor cells for 2 days. For *ex vivo* analysis from transplanted mice, T cells were cultured *ex vivo* with the indicated number of irradiated donor cells <24 h. For *in vitro* culture of OTI CD8^+^ T cells, 1×10^5^ purified OTI T cells/well were cultured with 2×10^4^/well irradiated (30Gy) aged or young splenic DCs in the presence of OVA peptide at the indicated dose for 72 h. Thymidine incorporation assay was used to measure cellular proliferation according to our prior work [27].

### ELISPOT and ELISA

ELISPOT analysis was performed as per our previously published work [26]. ELISA of culture supernatants was performed using reagents from BD Biosciences (San Diego, CA), according to manufacturer's instructions.

### Adoptive transfer of cells and cellular immunizations

5×10^5^ OTI (CD45.1^+^) and 5×10^5^ OTII (Thy1.1^+^) were adoptively transferred via i.v. tail vein injection into either aged or young syngeneic CD45.2^+^, Thy1.2^+^ C57BL/6 mice. Two days after adoptive transfer, mice received a skin graft from a B6.Act-mOVA donor. The absolute number of OTI/OTII T cells was calculated by multiplying the proportion of positive cells by the total number of splenocytes. Mice were immunized with 1×10^7^ BMDCs via i.p. injection. For immunization with allogeneic splenic DCs, mice received 3×10^5^ splenic DCs via i.v. tail vein injection. For adoptive transfer of syngeneic splenic DCs, pulsed with OVA_257–264_ peptide, DCs were 1^st^ incubated overnight with 50 ng/ml LPS. The cells were then washed and pulsed with 10 g µ/ml of OVA peptide for 2 h at 37°C and then washed three times prior to adoptive transfer into young hosts. Splenic DCs pulsed with an irrelevant peptide (GP33) acted as control. 5×10^5^ splenic DCs were transferred via i.v. tail vein injection along with 5×10^5^ CD45.1^+^ OTI T cells. Five days later, spleen cells were harvested from adoptively transferred mice for flow cytometric analysis.

### Cell sorting and flow cytometry

CD4, CD8, CD45.1, CD25, CD27, CD28, ICOS CD62L, Thy1.1/1.2, CD45.1, IL-2, TNFα and IFN-γ fluorescently labeled monoclonal antibodies and isotype controls were purchased from eBiosciences (San Diego, CA). Intracellular cytokine staining was achieved by harvesting spleen cells post transplantation and stimulating the cells ex-vivo with cognate peptide (for OTI and OTII TCR transgenic T cells) in the presence of cell permeability agents and golgi stop (eBiosciences). T cells were identified during the MLR by staining with Thy1.2 fluorescently labeled monoclonal antibody. All analysis was performed on a FACS CALIBUR flow cytometer and analyzed with Flow Jo software.

### Statistical analysis

Survival analysis between groups was calculated using the Log-rank method. Comparison of means was performed using a 2-tailed T-test and repeated measures using analysis of variance. All results were generated using GraphPad prism software (San Diego, CA). Statistical significance was considered a p value <0.05.
